# The Impact of Bariatric Surgery on Type 2 Diabetes Mellitus and the Management of Hypoglycemic Events

**DOI:** 10.3389/fendo.2017.00037

**Published:** 2017-03-01

**Authors:** Mahmoud Attia Mohamed Kassem, Michael Andrew Durda, Nicoleta Stoicea, Omer Cavus, Levent Sahin, Barbara Rogers

**Affiliations:** ^1^Department of Anesthesiology, The Ohio State University Wexner Medical Center, Columbus, OH, USA

**Keywords:** bariatric surgery, Roux-en-Y gastric bypass, post-bariatric surgery hypoglycemia, glucagon-like peptide-1, type 2 DM, C-peptide

## Abstract

Recent studies discussed the benefit of bariatric surgery on obese patients diagnosed with type 2 diabetes mellitus (T2DM). Several factors play an essential role in predicting the impact of bariatric surgery on T2DM, such as ABCD score (age, BMI, C-peptide, and duration of the disease), HbA1c, and fasting blood glucose, incretins [glucagon-like peptide-1 (GLP-1) and gastric inhibitory peptide (GIP)]. DiaRem score known to include factors such as age, HbA1c, medication, and insulin usage used to predict the remission of T2DM, but it has some limitations. An extensive literature search was conducted on PubMed and Google Scholar using keywords such as gastric bypass, T2DM, bariatric surgery, GLP-1, GIP, and post bariatric hypoglycemia. Restrictive-malabsorptive procedures are most effective in treating T2DM patients based on changes induced in appetite through regulation of gastrointestinal hormones, with decreased hunger and increased satiation. We provide a review of bariatric surgery influence on T2DM and management of post-intervention hypoglycemic events. Post-bariatric surgery hypoglycemia is a serious complication especially when patients develop life-threatening neuroglycopenia with loss of consciousness and seizure. The avoidance of this adverse event may be achieved by strict dietary modification including a restriction on carbohydrates as well as foods with high glycemic index. Further research will provide more information on post-bariatric surgery hyperinsulinemic hypoglycemia pathophysiology and management.

## Introduction

Obesity is one of the 10 leading US health indicators of increased risk of pulmonary diseases, cardiovascular diseases, diabetes, musculoskeletal diseases, and certain forms of cancers. The increasing prevalence of obesity in adult population triggered a concomitant rise in type 2 diabetes mellitus (T2DM) (1, 2). Obesity and insulin resistance are the main components of metabolic syndrome and result in impaired glucose metabolism. Those who suffer from both conditions are recently referred to as “diabesity patients” (3). Different obesity treatment strategies have been used, including prevention, lifestyle and dietary modification, behavioral therapy, and pharmacotherapy (1). A recent article published by Patti and Goldfine concluded that current therapies are not effective in providing a sustained weight loss (4). Of the various treatment options, bariatric surgery remains the most effective method to achieve a long-term weight loss (5, 6). Post-surgical weight loss improves all obesity-related comorbidities with a good quality of life and decreased overall mortality rate (7–9). Also, post-bariatric surgical patients have demonstrated 83% reduction in diabetes incidence, 30–40% reduction in myocardial infarction and stroke, 42% reduction of cancer in women, and 30–40% reduction in overall mortality (4). There are several bariatric procedures currently available including laparoscopic adjustable gastric banding (LAGB), laparoscopic sleeve gastrectomy (LSG), the biliopancreatic diversion with duodenal switch (LBPD-DS), and laparoscopic Roux-en-Y gastric bypass (LRYGB) (10).

PubMed and Google Scholar were searched for relevant articles. The following keywords and phrases were used in various combinations: gastric bypass, sleeve gastrectomy, T2DM, laparoscopic bariatric surgery, GLP-1, GIP, and post bariatric hypoglycemia. All articles identified within the initial search were screened for relevance and content, and their bibliographies were searched for any additional relevant articles. The criteria for inclusion were articles considering T2DM remission after bariatric surgery and post-bariatric surgery hypoglycemia (PBH). All publications up to June 2016 were considered.

## Bariatric Procedures

Laparoscopic adjustable gastric banding was the first bariatric technique to be performed by a laparoscopic approach. The operation is done by creation of a small pouch in the upper part of the stomach with a controlled and adjustable stoma, without stapling, thus limiting food intake. The band is fitted around the fundus of the stomach, forming a 15–20 ml small pouch. The diabetes remission after LAGB can be achieved gradually and is associated with the degree of weight loss (11).

Laparoscopic sleeve gastrectomy is a simple surgical technique with a low complication rate and minor long-term nutritional deficiencies. The operation is performed by resecting the greater curvature from the distal antrum (4 cm proximal to the pylorus) to the angle of His, including the complete fundus, by using a laparoscopic stapler. The remnant stomach tube was approximately 2 cm wide along the less curved side. The resected portion of the stomach was extracted from the extended periumbilical trocar site (12). A prospective study done by Silecchia et al. included 41 super-obese patients demonstrated that at 18th month post SG, diabetes was cured in 76.9% and improved in 15.4% of patients (13).

LBPD-DS, in this operation, is a 60% distal gastric resection with stapled closure of the duodenal stump results in a residual stomach volume of ~300 ml. The small bowel is transected 2.5 m from the ileocecal valve, and its distal end is anastomosed to the remaining stomach. The proximal end of the ileum, comprising the remaining small bowel carrying the biliopancreatic juice and excluded from food transit, is anastomosed to the bowel 50 cm proximal to the ileocecal valve. Consequently, the total length of absorbing bowel is 250 cm, the final 50 cm of which represents the site where ingested food and biliopancreatic juices mix (14).

Laparoscopic Roux-en-Y gastric bypass surgery, a restrictive and malabsorptive technique, is performed frequently in Canada and the US (112,000 procedures annually) (15, 16). In Europe, this technique is increasingly popular, with more than 26,000 procedures performed annually (16). A gastric pouch is created by completely separating stomach from the gastric remnant and anastomosed to the jejunum. An entero–entero anastomosis is created between the alimentary limb and pancreatobiliary limb, 75–150 cm distally from the gastrojejunostomy. The intake of food is restricted by gastric pouch and nutrients absorption is reduced by bypassing the duodenum and part of the jejunum (17). A recent study published in 2015 done by Yska et al. examining T2DM remission rate in patients who underwent bariatric surgery compared with T2DM patients without surgery demonstrated that LAGB had a smaller effect on T2DM remission than LRYGB and LSG (18).

## Preoperative Predictive Factors of T2DM Remission

Numerous studies have established the effect of bariatric surgery on remission or improvement of T2DM can be predicted by considering a number of factors (14, 19). A prospective cohort study published in 2014 by Itariu et al. concluded that the ABCD score (age, BMI, C-peptide, duration of the disease, also called the diabetes surgery score) is a simple multifactorial grading system that can predict the success of bariatric surgery on T2DM remission (see Table 1). The score contains four hubs, the degree of obesity (BMI), islet cell mass (C-peptide level), competence of B-cell function (T2DM duration), and the general leverage of body function (patient age) (20). The benefit of bariatric surgery in diabetic patients with BMI < 27 kg/m^2^ is reduced, and the procedure is not recommended for diabetes treatment (21, 22). Since chronic T2DM damages a large percentage of B-cells, patients with a short history of the disease are known to experience a greater benefit from surgery compared to those with a longer history of illness (22). C-peptide level is considered a mirror of insulin secretion, indicating the percentage of insulin secretion, B-cell functions, and surgical effect on T2DM remission (23). Patient age, *per se*, is considered to be of little value, but may predict the general physiological outcome, advanced age patients reporting minor benefits from undergoing bariatric surgery (24). Lee et al. compares the ABCD score (Table 1) with DiaRem score (Table 2) known to include factors as age, HbA1c, medication, and insulin usage. They suggested that the ABCD score is superior to DiaRem score, due to the limitations encountered by the latter. DiaRem score does not reflect the duration of T2DM (an example of the limitations), which is often considered the crucial predicting factor of diabetes remission.

**Table 1:** **ABCD grading system used to predict the success of bariatric surgery on type 2 diabetes mellitus (T2DM) remission**.

Factor	Score
**Age (years)**	
<40	1
≥40	0
**BMI (kg/m^2^)**	
<27	0
27–34.9	1
35–41.9	2
≥42	3
**C-peptide (ng/ml)**	
<2	0
2–2.9	1
3–3.9	2
≥5	3
**Duration of DM (years)**	
>8	0
4–8	1
1–3.9	2
<1	3
Total score calculated by adding each of the four variables	0–10

**Table 2:** **DiaRem grading system used to predict the success of bariatric surgery on type 2 diabetes mellitus (T2DM) remission**.

Factor	Score
**Age (years)**	
<40	0
40–49	1
50–59	2
≥60	3
**HbA1c (%)**	
<6.5	0
6.5–6.9	2
7–8.9	4
≥9.0	6
**Other diabetes drugs**	
No sulfonylureas or insulin-sensitizing agents other than metformin	0
Sulfonylureas and insulin-sensitizing agents other than metformin	3
**Treatment with insulin**	
No	0
Yes	10
Total score calculated by adding each of the four variables	0–22

Furthermore, DiaRem score was developed based on studies enrolling patients with a mean BMI of 48.8 kg/m^2^ undergoing bariatric surgery regardless diabetes status (25).

## Mechanisms of T2DM Remission after Bariatric Surgery

Several studies suggested that a decrease in plasma glucose level in T2DM patients after bariatric surgery is a result of a caloric restriction, not of a significant weight loss. Even though any decrease in caloric intake can improve plasma glucose levels and liver fat content, the mechanism of T2DM remission after bariatric surgery is still not fully elucidated (26). Numerous studies postulated that intestinal hormonal changes after bariatric surgery play an important role in T2DM remission and developed two hypotheses: the hindgut hypothesis and the foregut hypothesis. The hindgut hypothesis of T2DM remission proposes that following bypass surgery, there is a rapid delivery of nutrients to the distal intestine stimulating L-cell secretion of anorexigenic and antidiabetic peptides, including GLP-1 and peptide YY (27, 28). This hypothesis focuses on GLP-1 because its effects on B-cells proliferation and insulin production (29). The foregut hypothesis states that bypassing the proximal small intestine the secretion of anti-incretin hormones is diminished and blood glucose control is improved (28). Gastric inhibitory peptide (GIP) is another incretin responsible for increased postprandial insulin release after bypass surgery (30).

Ghrelin is a 28-amino acid polypeptide hormone produced mainly by endocrine A-like cells in the gastric and duodenum epithelia being responsible for appetite stimulation; its concentration is reduced after bypass surgery as a result of lack of food stimulus (31).

Changes are also seen in adipocyte-derived hormones, leptin is correlated with insulin resistance whereas adiponectin enhances insulin sensitivity (32). After bypass surgery, a decrease in leptin level and a rise in adiponectin concentration are noted (33, 34). A recently published study investigating adipocyte-derived exosomal microRNAs after gastric bypass showed that there are major changes in these microRNAs which correlated to improvement of insulin resistance and metabolomic changes consistent with improved glucose homeostasis (35).

A randomized controlled trial comparing RYGB to a lifestyle intervention included Taiwanese and American patients published in 2016 by Chong et al. found that RYGB was associated with better improvement and remission of diabetes in both ethnicities (36).

As the aforementioned findings indicate, success of T2DM *via* bariatric surgery can be predicted and that the actual mechanism supporting these phenomena appears multifactorial and may involve changes in gut- and adipocyte-derived hormones (37).

## PBH and Its Management

One of the severe complications of Roux-en-Y gastric bypass surgery is PBH, first described by Service et al. in 2005 and subsequently confirmed by other authors (38–40). PBH is defined as a delayed onset hypoglycemia (1–2 h after a meal) without vasomotor symptoms. This presentation differs from early dumping syndrome characterized by fast onset (10–30 min after food intake) due to rapid food entrance into the intestine, and accompanied by an increased osmotic effect, with a fall in blood pressure and rise in heart rate (41).

Hypoglycemic symptoms can be classified as autonomic (such as palpitations, lightheadedness, sweating, anxiety, tremors, hunger) or neuroglycopenic (such as fainting, dizziness, blurred vision, weakness, confusion, decreased attentiveness, seizure, loss of consciousness). Autonomic symptoms occur when the glucose level drops below 3.3 mmol/l (60 mg/dl), and neuroglycopenic symptoms predominate when the level drops below 2.8 mmol/l (50 mg/dl) (42). Hypoglycemic symptoms experienced early in the postoperative period are often associated with dumping syndrome and are effectively treated with low glycemic index diets. More severe hypoglycemia associated with neuroglycopenia is manifested as a loss of consciousness, seizure activities, and typically reported 1–3 years after gastric bypass (4). To confirm that symptoms are related to hypoglycemia, venous blood sampling should demonstrate glucose values <70 mg/dl (3.9 mmol/l), and fast improvement of symptoms after glucose ingestion. Also, plasma insulin concentrations are inappropriately high at the time of hypoglycemia, indicating dysregulation of insulin secretion as an important mechanism (4). The published prevalence of hypoglycemia following bariatric surgery varies, depending on the applied definition, with a range of 0.2% for patients requiring hospitalization for severe hypoglycemia to 72% for reactive hypoglycemia after a glucose tolerance test (43, 44). A better understanding of hypoglycemia mechanism in post-bariatric surgery patients will improve the glycemic control management (39, 45). A common hypothesis for PBH supports the evidence that increased secretion and action of GLP-1 enhances the cell response to meal ingestion, entertaining hypoglycemic events (40). GB recipients present higher plasma levels of GLP-1 and greater GLP-1 action to increase insulin release than individuals who did not require this intervention. According to studies testing GLP-1 receptor antagonists (exendin-9) during meal ingestion, no difference was reported on postprandial GLP-1 receptor antagonist action on hypoglycemic versus normoglycemic individuals (46). Service et al. proposed a possible mechanism responsible for the development of PBH due to islet cell hyperplasia with amplification of beta cell mass that contribute to increased insulin level and subsequent hypoglycemia (39). Other potential mechanisms have been suggested such as altered intestinal microbiota, augmented bile acids, and enhanced glucose efficacy (4, 47). Thus, the mechanisms of postprandial hypoglycemia in GB subjects are complex remain unknown (40). Therapeutic approaches to prevent hypoglycemia following RYGB are not standardized. Medical treatment is directed toward maintaining blood glucose levels within normal limits (48). PBH patients usually develop a postprandial high glucose level following carbohydrate-enriched food consumption stimulates GLP-1 and insulin release, with a rapid drop of glucose level and subsequent hypoglycemia. A carbohydrate restrictive diet “high glycemic index food and simple sugars” address symptoms and prevents hypoglycemia and hyperinsulinism (49–51). Other dietary interventions such as increased dietary fiber intake and avoiding alcohol consumption are responsible for a decrease in glucose absorption and improved postprandial hypoglycemia (52–55). Bantle et al. and Tappy and Mittendorfer demonstrated that fructose can be ingested safely by PBH patients, with avoidance of over consumption and undesirable effect on metabolic health (56, 57). McLaughlin et al. suggested that post RYGB patients with neuroglycopenic symptoms improved after gastrostomy and continuous enteral feeding with decreased glucose and insulin levels and significantly lower values of GLP-1, GIP, and glucagon when compared with previous oral nutrition (58). Medications, such as acarbose, diazoxide, octreotide, or verapamil have been known to successfully treat this unexpected surgical outcome (1, 59). Acarbose is effective in reducing blood sugar level, plasma insulin levels, and GLP-1 (60). Common side effects associated with acarbose were bloating, flatulence, or diarrhea (10). Combination of verapamil with acarbose was found to be effective in PBH (61). Hypotension and edema are common side effects associated with verapamil use (10). Diazoxide inhibits insulin release through the activation of ATP-sensitive potassium channel (62). Diazoxide use may be responsible for hypotension, fluid retention, and hirsutism (10). Octreotide is advised when diet, α-glucosidase, and calcium channel blockers have not been effective (63). Invasive procedures, for instance restoring gastric restriction with an adjustable gastric band, placing gastrostomy tube, subtotal, or total pancreatectomy, are considered as a part of the treatment (1, 58, 59, 64). However, preoperative individual risk assessment is required as long as factors predicting PBH following bariatric surgery are still investigated (61).

## Conclusion

Diabetes remission, improvement of blood glucose control, and reduction of antidiabetic medications after bariatric surgery can be sustained for many years with a decrease in overall morbidity and mortality. There are grading systems to predict T2DM remission success and that while the mechanisms supporting this are not fully understood there are a number of hormonal changes that may play a significant role in stabilizing blood glucose levels, improving insulin sensitivity, and regulating appetite which overall have a beneficial effect on T2DM. Restrictive-malabsorptive procedures are known to improve glucose homeostasis, insulin sensitivity, and B-cells secretory function. Bariatric surgery can be considered as a second-line therapy in non-obese diabetic patients. In non-diabetic morbidly obese patients, bariatric surgery may prevent the development of T2DM and other comorbidities. PBH is a serious complication, dysregulation of insulin secretion with hyperinsulinemic state being the most important predicting factor for this group of surgical patients. Food restriction is the first-line therapy for this condition and if failed, pharmacological management should be considered. Pancreatectomy may be required for limited cases. Further research should identify the patient population at risk for post-bariatric hypoglycemia and elaborate on the effective treatment. The metabolic benefits, as well as short- and long-term surgical complications, should be considered when the patient is advised to proceed with surgery.

## Author Contributions

MK, MD, NS, OC, and LS conducted a review of the literature. MK, MD, NS, OC, and LS prepared the body of the manuscript. BR critically reviewed the publication. All the authors endorsed the final form of the manuscript.

## Conflict of Interest Statement

The authors declare that the research was conducted in the absence of any commercial or financial relationships that could be construed as a potential conflict of interest.
